# KISS1 Suppresses Apoptosis and Stimulates the Synthesis of E2 in Porcine Ovarian Granulosa Cells

**DOI:** 10.3390/ani9020054

**Published:** 2019-02-12

**Authors:** Xiaoping Xin, Zhonghui Li, Yuyi Zhong, Qingqing Li, Jiaying Wang, Hao Zhang, Xiaolong Yuan, Jiaqi Li, Zhe Zhang

**Affiliations:** Guangdong Provincial Key Lab of Agro-Animal Genomics and Molecular Breeding, National Engineering Research Centre for Breeding Swine Industry, College of Animal Science, South China Agricultural University, Guangzhou, Guangdong 510642, China; xiaopingxin1991@163.com (X.X.); lizh_scau@163.com (Z.L.); yyzhong04@163.com (Y.Z.); qingqingli87@163.com (Q.L.); jiayingwang1993@163.com (J.W.); zhanghao@scau.edu.cn (H.Z.); yxl@scau.edu.cn (X.Y.); zhezhang@scau.edu.cn (Z.Z.)

**Keywords:** *KISS1*, Granulosa cells, Cell apoptosis, Cell cycle, Synthesis of E2

## Abstract

**Simple Summary:**

In mammals, KISS-1 metastasis suppressor (*KISS1*) has emerged to stimulate the secretion of gonadotropin-releasing hormone (GnRH) to initiate the first estrus in the hypothalamus. However, *KISS1* was recently demonstrated to be widely expressed in various ovarian compartments, including oocytes, granulosa cells (GCs) and theca cells. But the biological functionalities of *KISS1* have not been explored in GCs. In this study, the overexpression plasmid of pcDNA3.1-KISS1 was built to explore the biological effects of *KISS1* on the phosphoinositide 3-kinases (PI3K) signaling pathway, estrogen signaling pathway, cell apoptosis, cell cycle, and estradiol-17β (E2) secretion in porcine GCs. We found that overexpression of *KISS1* could affect the PI3K signaling pathway, significantly decrease the apoptosis of GCs, and suppress GCs at G0/G1 phase of the cell cycle. Furthermore, overexpression of *KISS1* could activate the estrogen synthesis signaling pathway and significantly increase the concentration of E2 in the supernatant and the mRNA expression levels of *ESR1* and *ESR2.* These findings were highly accorded with the supposed role of *KISS1* to promote the follicular development. This study would be of great interest for exploring the biological functionalities of *KISS1* in regulating the maturation of follicles in mammals.

**Abstract:**

Previous studies have strongly recommended that KISS-1 metastasis suppressor (*KISS1*) plays an essential gatekeeper of the initiation of reproductive maturation in mammals. However, *KISS1* has been recently reported to highly express in ovarian granulosa cells (GCs). But the biological functionalities of *KISS1* on cell apoptosis, cell cycle, and synthesis of estradiol-17β (E2) have not been explored in GCs. In this study, using porcine GCs as a cellular model, the overexpression plasmid of *KISS1* was built to explore the biological effects of *KISS1* on the PI3K signaling pathway, estrogen signaling pathway, cell apoptosis, cell cycle, and E2 secretion. We found that mRNA of *KISS1* highly expressed in the ovary and significantly increased from immature to mature follicles in gilts. Overexpression of *KISS1* could significantly increase the mRNA expression of *PIK3CG, PIK3C1,* and *PDK1*, and significantly decreased the mRNA levels of *FOXO3*, *TSC2*, and *BAD* of PI3K signaling pathway. Furthermore, results of the flow cytometry showed that overexpression of *KISS1* significantly inhibited the apoptosis of GCs and decreased the percentage of GCs at G0/G1 phase of the cell cycle. Additionally, overexpression of *KISS1* could increase the mRNA levels of *Star*, *CYP17*, *3B-HSD*, *17B-HSD* of estrogen synthesis signaling pathway, significantly increase the concentration of E2 in the supernatant of the cultured GCs, and up-regulate the mRNA expression levels of *ESR1* and *ESR2*. These results suggested that *KISS1* might suppress cell apoptosis through activating the PI3K signaling pathway and stimulate synthesis of E2 via boosting the estrogen synthesis signaling pathway. This study would be of great interests for exploring the biological functionalities of *KISS1* in the folliculogenesis and sex steroid production of the ovaries in mammals.

## 1. Introduction

In many mammalian species, the initiation of first estrus indicating the sexual and reproductive maturation is activated as a result of an increase in the gonadotropin releasing hormone (GnRH) secretion [1,2]. Kisspeptins, the products of *KISS1* gene, have recently emerged as an essential gatekeeper for the onset of first estrus via directing the stimulation of GnRH secretion at the hypothalamic level [3,4,5]. Recent studies have shown that *KISS1* are widely expressed in the ovarian tissues [6,7], indicating its additional local function in reproduction at the extra-hypothalamic level. In gene knockout models, *KISS1*^−/−^ [8,9] or *KISS1 receptor* (*KISS1R*)^−/−^ [10,11] mice show small ovarian size and weight, compared to the wild-type counterparts. In cats, *KISS1* and *KISS1R* are expressed in various ovarian compartments, including oocytes, granulosa cells (GCs) and theca cells [6,12]. Compared with the theca cells and other ovarian cells, previous studies report that *KISS1* mRNA expression is significantly higher in the GCs and suggest that GC is the major site for kisspeptin synthesis in ovaries of mammals [6,13]. These results suggest the proposal and essential role of *KISS1* in ovaries of mammals.

In mammals, it is widely known that GCs play a vital regulatory role in deciding the fate of follicles and the follicular maturation [14,15,16]. Furthermore, estrogens produced in GCs are suggested to support the survival and proliferation of GCs [17,18] and facilitate the maturation of follicles [19,20], which are mainly regulated and controlled by phosphatidylinositol 3-OH-kinase (PI3K) signaling pathway [21,22,23]. Recently, the mRNA level of *KISS1* has been found to be significantly upregulated in human granulosa lutein cells obtained from women with polycystic ovary syndrome [24,25], and *KISS1* has been suggested to stimulate progesterone in GCs of pigs [26]. However, the biological functions of *KISS1* on cell survival and steroidogenesis were still unknown in mammalian GCs.

In this study, the expression changes of *KISS1* mRNA were first detected between immature to mature follicles in pigs. Using porcine GCs as a cellular model, the overexpression plasmid of *KISS1* was then built to explore the biological effects of *KISS1* on the PI3K signaling pathway, estrogen signaling pathway, cell apoptosis, cell cycle, and estradiol-17β (E2) secretion. To the best of our knowledge, this study is the first time to explore the biological molecular functionalities of *KISS1* on cell survival and steroidogenesis in mammalian GCs.

## 2. Methods and Materials

### 2.1. Ethics Approval

All experiments in the present study were performed in accordance with the guidelines of the Animal Care and Use Committee of South China Agricultural University Guangzhou, China (Approval Number: SCAU#2013-10).

### 2.2. Animals and Sample Preparation

In female pigs, the follicular maturation could be handily identified by the first standing reflex with the back-pressure test and boar contact [27]. Three Landrace × Yorkshire crossbred gilts at the day that they exhibited the first estrus and standing reflex were selected. The signs of first estrus were checked and recorded twice daily at 09:00 and 15:30 by inspection of the vulva and assessment of the standing reflex according to other studies [28,29]. The tissues of heart, liver, spleen, lung, cerebrum, cerebellum, pituitary, and ovary were collected from these pigs. At least three of the largest follicles (8–10 mm) of the unilateral ovaries from these gilts were collected as the mature follicles, and as least three of follicles within the length of 5–7 mm was collected as the immature follicles. Pigs were fed the same diet daily and reared in the same conditions and environments. The collected follicles were frozen quickly in liquid nitrogen and then stored at −80 °C for further use.

### 2.3. Culture of Porcine GCs In Vitro

The porcine ovarian GCs were cultured as previously described in our studies [30,31]. Briefly, ovaries were collected from a local slaughterhouse for pigs in Guangzhou and transferred to our laboratory in phosphate-buffered saline (PBS) containing penicillin (100 IU/mL) and streptomycin (100 μg/ mL) (Invitrogen, Shanghai, China) at a storage temperature of >30 °C. Subsequently, 5–7 mm follicles were punctured for GC collection using a 1-mL syringe, and the isolated GCs were washed twice with PBS preheated to 37 °C. The cells were seeded into 25-cm^2^ flasks and cultured at 37 °C under 5% CO_2_ in DMEM (Hyclone, Logan, UT, USA) containing 10% fetal bovine serum (Hyclone), 100 IU/mL penicillin, and 100 μg/mL streptomycin. When cells reached 80% coverage of the flask, cells were seeded into 24-well plates for further experiments.

### 2.4. Real-Time Quantitative PCR Analysis

When the cells reached 30–50% coverage of one well, pcDNA3.1-KISS1 and pcDNA3.1-Basic were transfected into the cells at 200 ng for 48 h, respectively. At least 3 wells per group was collected for extraction of total RNA. Total RNA was extracted using TRIzol reagent (TaKaRa, Tokyo, Japan) and then reverse-transcribed using a RevertAid First Strand cDNA Synthesis Kit (Thermo Scientific, Waltham, MA, USA) for mRNAs. The relative expression levels of mRNAs were quantified using Maxima SYBR Green qRT-PCR Master Mix (2×) (Thermo Scientific) -in a LightCycler Real-Time PCR system. The expression levels of *GAPDH* mRNAs were used as endogenous controls, and the fold changes were calculated using the 2^−ΔΔct^ method. The primer sequences are listed in Table 1.

### 2.5. Cell Apoptosis Assay

According to the NCBI database, the coding sequences of *KISS1* (Gene ID: 100145896, Accession Number: NM_001134964.1) were cloned into pcDNA3.1 (+) (ThermoFisher, Guangzhou, China) with the restrictive enzymes of EcoRI and NotI. The pcDNA3.1-KISS1 and pcDNA3.1-Basic were transfected in GCs by using Lipofectamine^TM^ 3000 Transfection Reagent (ThermoFisher).

Analysis and detection of cell apoptosis were referred to in one of our previous studies [32]. Cell apoptosis assays were performed using an Annexin V-FITC Apoptosis Detection Kit (BioVision, Milpitas, CA, USA) according to the manufacturer’s instructions. Briefly, GCs (1–5 × 10^5^ cells/well) were cultured in triplicate in 6-well plates at one day prior to transfection. When the cells reached 30–50% coverage of one well, pcDNA3.1-KISS1 and pcDNA3.1-Basic were transfected into the cells at 200 ng for 48 h, respectively. The cells were then harvested, washed twice with ice-cold PBS, and resuspended in 500 μL of binding buffer. Next, 1.25 μL of Annexin V-FITC was added in the dark for 15 min at room temperature, then 1000× *g* centrifugation for 5 min at room temperature to remove the supernatant. The cells were gently resuspended with 0.5 mL precooling 1× solution, and 10 μL of PI (propidium iodide; 50 μg/mL, BD, New York, NY, USA) were added. Last, the cells were analyzed in a flow cytometer (Becton Dickinson Co., San Jose, CA, USA) using the FITC signal detector (FL1) and phycoerythrin emission signal detector (FL2). All experiments were performed at least three times. Cells in the lower right quadrant are annexin-positive/PI-negative early apoptotic cells. The cells in the upper right quadrant are annexin-positive/PI-positive late apoptotic cells. Cells undergoing early and late apoptosis were identified as the apoptotic cells.

### 2.6. Cell Cycle Analysis

When the cells reached 30–50% coverage of one well, pcDNA3.1-KISS1 and pcDNA3.1-Basic were transfected into the cells at 200 ng for 48 h, respectively. The cells were then harvested, washed twice with ice-cold PBS, and resuspended with a propidium iodide/RNase A solution at 37 °C in a dark for 30 min. Then cells were analyzed by flow cytometry (Becton Dickinson Co., San Jose, CA, USA). For each analysis, a minimum of 20,000 cells were analyzed.

### 2.7. ELISA for Measurements of Steroid Hormones

After transduction with pcDNA3.1-KISS1 and pcDNA3.1-Basic for 48 h, the concentrations of E2 in the culture supernatants were measured with ELISA kits (Beijing north institute of biological technology, Beijing, China) according to the manufacturer’s instructions.

### 2.8. Data Analysis

All cell experiments were repeated at least three times independently. For cell apoptosis, the fold change of cell apoptosis rate was presented, compared to the blank group. Compared to the blank group, the fold change of the concentrations of E2 was exhibited. Data were expressed as means ± SD of repeated experiments. Statistical analyses were carried out using R software (version-3.4.3, https://www.r-project.org/). Tukey’s test was used to test whether the variances among the mRNA relative expression of *KISS1* in different tissues were significant. Student’s t-test (two-tailed) was used to analyze the significance of differences of two groups in data. * indicates *p* < 0.05; ** indicates *p* < 0.01.

## 3. Results

### 3.1. Expression of KISS1 during Follicular Maturation in Pigs

**Figure 1:**
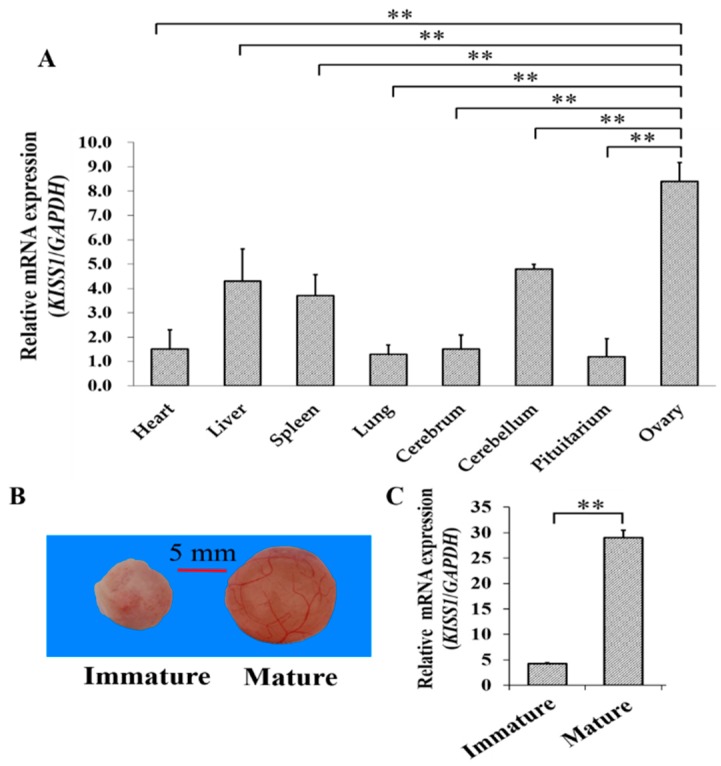
Relative mRNA Expression of *KISS1* during follicular maturation in pigs. (**A**) Tissue expression profile of *KISS1* mRNA in gilts with the first standing reflex. (**B**) Representation of immature to mature follicles. (**C**) Changes of *KISS1* mRNA expression from immature to mature follicles. ** indicates *p* < 0.01.

The mRNA expression pattern of *KISS1* was first explored for tissues from gilts with the first standing reflex (see Methods and Materials, Section 2.2. Animals and Sample Preparation). Among the tissues of heart, liver, spleen, lung, cerebellum, pituitarium, and ovary, we found that mRNA of *KISS1* expressed the highest in the ovary (Figure 1A). To further investigate the biological role of *KISS1* during the follicular maturation, the mRNA expression levels of *KISS1* were examined in porcine follicles from immature to mature (see Methods and Materials, Section 2.2. Animals and Sample Preparation) (Figure 1B), and mRNA expression levels of *KISS1* in mature follicles were observed to be significantly higher than immature follicles (Figure 1C). These observations indicated that *KISS1* might be involved in the processes of follicular mature in pigs.

### 3.2. Biological Effects of KISS1 on Cell Apoptosis and Cell Cycle of GCs

**Figure 2:**
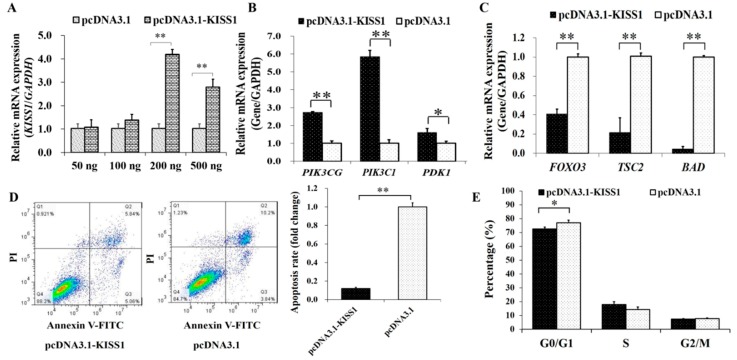
Biological Effects of KISS1 on cell apoptosis and cell cycle of GCs. (**A**) Relative mRNA expressions of KISS1 against the different concentrations of pcDNA3.1-KISS1 plasmid. (**B**) Relative mRNA expressions of *PIK3CG*, *PIK3C1*, and *PDK1* were stimulated by pcDNA3.1-KISS1. (**C**) Relative mRNA expressions of *FOXO3*, *TSC2*, and *BAD* were suppressed by pcDNA3.1-KISS1. (**D**) PcDNA3.1-KISS1 decreased cell apoptosis. (**E**) Effects of pcDNA3.1-KISS1 on the percentage of different cell cycle stage. * indicates *p* < 0.01. ** indicates *p* < 0.01. Data were represented as means ± SD.

The overexpression plasmid of *KISS1* were then built to explore the effects of *KISS1* on PI3K signaling pathway, apoptosis and cycle in GCs (Figure 2). We found that the mRNA expression of *KISS1* was the highest at the 200 ng of pcDNA3.1-KISS1 (Figure 2A), and the 200 ng of pcDNA3.1-KISS1 plasmid was selected and used for further analysis. Compared to the control group, we found pcDNA3.1-KISS1 could significantly increase the mRNA expression levels of *PIK3CG*, *PIK3C1* and *PDK1* (Figure 2B), but pcDNA3.1-KISS1 significantly decreased the mRNA expression levels of *FOXO3*, *TSC2* and *BAD* (Figure 2C).

Furthermore, the annexin V-FITC flow cytometry was used to analysis the cell apoptosis, and the apoptosis rate of GCs in pcDNA3.1-KISS1 group was observed to be significantly lower than the control group (Figure 2D). Analysis of cell cycle showed that pcDNA3.1-KISS1 significantly decreased the percentage of cells in G0/G1 (Figure 2E).

### 3.3. Biological Effects of KISS1 on Synthesis of Estrogen in GCs

**Figure 3:**
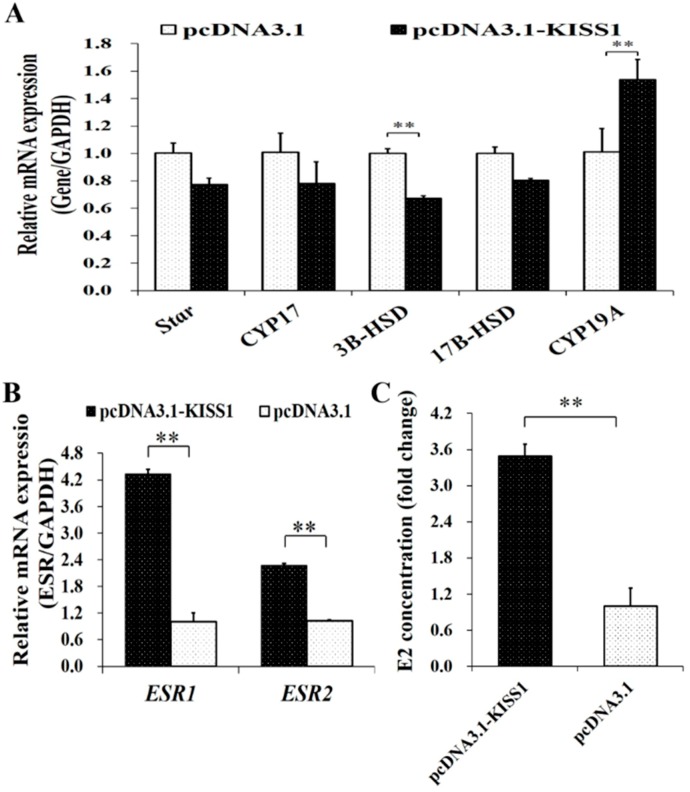
Biological Effects of *KISS1* on synthesis of estrogen in GCs. (**A**) Effects of pcDNA3.1-KISS1 on the relative mRNA levels of *Star*, *CYP17*, *3B-HSD*, *17B-HSD*, and *CYP19A*. (**B**) Concentrations of E2 was stimulated by pcDNA3.1-KISS1. (**C**) Effects of pcDNA3.1-KISS1 on the relative mRNA levels of *ESR1* and *ESR2*. ** indicates *p* < 0.01. Data were represented as means ± SD.

To further validate the biological functions of *KISS1* on the synthesis of E2 in GCs, pcDNA3.1-KISS1 was transfected into porcine GCs. The mRNA expression of *Star*, *CYP17*, *3B-HSD*, *17BHSD* and *CYP19A* from the estrogen signaling pathway were first detected (Figure 3A). Compared to the control group, overexpression of *KISS1* could significantly increase mRNA expression levels of *CYP19A* but significantly decrease the mRNA expression levels of *3B-HSD* (Figure 3A). Moreover, overexpression of *KISS1* could significantly increase the concentrations of E2 in the culture supernatants (Figure 3B). Additionally, the mRNA expression changes of ESRs were also detected after the administration of pcDNA3.1-KISS1, and pcDNA3.1-KISS1 was observed to significantly increase the mRNA expression levels of *ESR1* and *ESR2* (Figure 3C).

## 4. Discussion

Recently, although new insight on *KISS1* in ovaries has been described in several mammals [6,33,34], very little is known regarding the biological functions of *KISS1* in the ovarian tissues. *KISS1* has been suggested to highly express in GCs [6,13] and might play a significantly regulatory role in regulating follicular maturation in mammals [6,7,35]. In this study, we found that mRNA level of *KISS1* expressed relatively highly in the ovary (Figure 1A) and significantly increased from immature to mature follicles (Figure 1B,C).

Most importantly, the relative mRNA level of *KISS1* is significantly higher in the follicular stage than in the luteal stage [6]. In rats, *KISS1* mRNA expression is changed in a cyclic-dependent manner in the ovary, with a rise during the preovulatory period [13,36]. In cows, compared to control group, administration of kisspeptin, the product of *KISS1*, can accelerate the follicular growth and increase the follicular sizes of the dominant follicles [34]. In mice, *KISS1* has been identified as one of the differentially expressed genes of ESR2-mutant young adult females with the failure of follicular maturation [37]. In humans, compared to the normal women, the infertile women with polycystic ovary syndrome have increased serum kisspeptin levels [38], and serum kisspeptin has a positive correlation with the serum levels of LH and leptin [39]. These observations suggest that ovarian *KISS1* may play a critical role in regulating the follicle maturation.

The PI3K signaling pathway is widely reported to be crucial for follicle growth [40] and promotes cell survival and suppress apoptosis in mammalian GCs [21,23]. Most importantly, the growth and proliferation of GCs play critical roles in the biological processes of recruitment, selection and maturation of follicles [20,41]. In this study, we found that pcDNA3.1-KISS1 could significantly increase the mRNA expression of *PIK3CG*, *PIK3C1*, *PDK1* (Figure 2B), which are the important upstream genes of the PI3K signaling pathway. But pcDNA3.1-KISS1 significantly decreased the mRNA levels of *FOXO3, TSC2, and BAD* (Figure 2C), which are the important downstream genes of the PI3K signaling pathway. Previous studies recommend that *PIK3CG* exhibits significant DNA copy number gains in ovarian cancer, compared to normal ovary in humans [42], *PIK3C1* promotes the activation of primordial follicles [43], and moreover, in *PDK1* depletion in oocytes depletes the majority of primordial follicles around the onset of sexual maturity and causes premature ovarian failure in mice [44]. In addition, *FOXO3* depletion in GCs disrupts the normal ovarian follicular growth in mice [45], and a lower FOXO3 mRNA expression in GCs may lead to infertility in women [46]. *BAD* can reduce progesterone levels by promoting ovarian GC apoptosis in sheep [47]. Being TSC2-deficient in oocytes results in the depletion of follicles in early adulthood [48]. Furthermore, results of Annexin V-FITC flow cytometry further identified that pcDNA3.1-KISS1 inhibited the apoptosis of GCs (Figure 2D). Moreover, pcDNA3.1-KISS1 was found to decrease the percentage of GCs at G0/G1 phase of the cell cycle (Figure 2E). These observations demonstrated that *KISS1* was likely to be involved in the PI3K signaling pathway, suppress cell apoptosis, and regulate cell cycle entry.

In mammalian ovaries, E2 was mainly synthetized and produced in GCs [49,50] and then to stimulate the differentiation of GCs [17,18] and facilitate the maturation of follicles [19]. In this study, we found that pcDNA3.1-KISS1 could significantly increase the mRNA levels of *3B-HSD* and *CYP19A* of estrogen synthesis signaling pathway (Figure 3A) and increase the concentration of E2 in the supernatant of the cultured GCs (Figure 3B). Moreover, pcDNA3.1-KISS1 could increase the mRNA expression levels of *ESR1* and *ESR2* (Figure 3C). These results suggested that *KISS1* might stimulate the synthesis of E2 in porcine GCs.

## 5. Conclusions

Collectively, *KISS1* was observed to involve in PI3K signaling pathway, suppress cell apoptosis, and stimulate synthesis of E2 via boosting the estrogen synthesis signaling pathway. Moreover, these findings were highly accorded with the supposed role of *KISS1* promoting the follicular mature. These results would be of great interest for exploring the biologically molecular functionalities of *KISS1* on cell survival and steroidogenesis in mammalian GCs.

## Figures and Tables

**Table 1:** Primers used in the present study.

Name	Sequence	Product (bp)	Accession Number
CDS-KISS1	F: GAATTCATGAATGCACTGGTTTTTTGG	431	NM_001134964.1
	R: CGCCGGCGAGTCAGAGCGGGCCGCGGAA		
qRT-PCR-KISS1	F: AACCAGCATCTTCTCACCAGG	192	NM_001134964.1
	R: CTTTCTCTCCGCACAACGC		
qRT-PCR-GAPDH	F: TCCCGCCAACATCAAAT	163	XM_021091114.1
	R: CACGCCCATCACAAACAT		
qRT-PCR-PIK3CG	F: AACGGGCTTTGAGATAGTGAA	184	NM_213939.1
	R: AAGTTGCTTGGTTGGTGGATA		
qRT-PCR-PIK3C1	F: CAAGTGAGAATGGTCCGAATG	152	NM_006218.3
	R: GTGGAAGAGTTTGCCTGTTTT		
qRT-PCR-PDK1	F: AAATCACCAGGACAGCCAATA	190	NM_001159608.1
	R: CTTCTCGGTCACTCATCTTCAC		
qRT-PCR-FOXO3	F: ACAAACGGCTCACTCTGTCCCA	85	NM_001135959.1
	R: GAACTGTTGCTGTCGCCCTTATC		
qRT-PCR-TSC2	F: CGAGGTGGTGTCCTACGAGAT	115	XM_005655162.3
	R: GAGCAGGCGTTCAATGATGTT		
qRT-PCR-BAD	F: AGTCGCCACTGCTCTTACCC	172	XM_021082883.1
	R: TCTTGAAGGAACCCTGGAAATC		
qRT-PCR-Star	F: GGAAAAGACACAGTCATCACCCAT	121	NM_213755.2
	R: CAGCAAGCACACACACGGAAC		
qRT-PCR-CYP17	F: AAGCCAAGACGAACGCAGAAAG	228	NM_214428.1
	R: TAGATGGGGCACGATTGAAACC		
qRT-PCR-3B-HSD	F: GGGGCTTCTGTCTTGATTCCA	284	NM_001004049.2
	R: GGTTTTCAGTGCTTCCTTGTGC		
qRT-PCR-17B-HSD	F: CCCAACGCAGGAGACTCAAAAT	149	NM_214306.1
	R: CCAGAGCCCATAACGAAGACAGA		
qRT-PCR-CYP19A	F: GCTGGACACCTCTAACAACCTCTT	91	NM_214430.1
	R: TTGCCATTCATCAAAATAACCCT		
qRT-PCR-ESR1	F: GATGCCTTGGTCTGGGTGAT	124	XM_003468423.2
	R: AGTGTTCCGTGCCCTTGTTA		
qRT-PCR-ESR2	F: AAGGGAAAAGGAGGATGGGACA	202	NM_0010011533.1
	R: CAGATAGGGACTGCGTGGAGGT		
